# Stimulation and Recording of the Hippocampus Using the Same Pt-Ir Coated Microelectrodes

**DOI:** 10.3389/fnins.2021.616063

**Published:** 2021-02-24

**Authors:** Sahar Elyahoodayan, Wenxuan Jiang, Curtis D. Lee, Xiecheng Shao, Gregory Weiland, John J. Whalen, Artin Petrossians, Dong Song

**Affiliations:** ^1^Department of Biomedical Engineering, Center for Neural Engineering, University of Southern California, Los Angeles, CA, United States; ^2^Epic Medical, Inc., Pasadena, CA, United States; ^3^Neuroscience Graduate Program, University of Southern California, Los Angeles, CA, United States

**Keywords:** Pt-Ir electrodeposition, intracortical stimulation, intracortical recording, electrochemistry, electrophysiology

## Abstract

Same-electrode stimulation and recording with high spatial resolution, signal quality, and power efficiency is highly desirable in neuroscience and neural engineering. High spatial resolution and signal-to-noise ratio is necessary for obtaining unitary activities and delivering focal stimulations. Power efficiency is critical for battery-operated implantable neural interfaces. This study demonstrates the capability of recording single units as well as evoked potentials in response to a wide range of electrochemically safe stimulation pulses through high-resolution microelectrodes coated with co-deposition of Pt-Ir. It also compares signal-to-noise ratio, single unit activity, and power efficiencies between Pt-Ir coated and uncoated microelectrodes. To enable stimulation and recording with the same microelectrodes, microelectrode arrays were treated with electrodeposited platinum-iridium coating (EPIC) and tested in the CA1 cell body layer of rat hippocampi. The electrodes’ ability to (1) inject a large range of electrochemically reversable stimulation pulses to the tissue, and (2) record evoked potentials and single unit activities were quantitively assessed over an acute time period. Compared to uncoated electrodes, EPIC electrodes recorded signals with higher signal-to-noise ratios (coated: 9.77 ± 1.95 dB; uncoated: 1.95 ± 0.40 dB) and generated lower voltages (coated: 100 mV; uncoated: 650 mV) for a given stimulus (5 μA). The improved performance corresponded to lower energy consumptions and electrochemically safe stimulation above 5 μA (>0.38 mC/cm^2^), which enabled elicitation of field excitatory post synaptic potentials and population spikes. Spontaneous single unit activities were also modulated by varying stimulation intensities and monitored through the same electrodes. This work represents an example of stimulation and recording single unit activities from the same microelectrode, which provides a powerful tool for monitoring and manipulating neural circuits at the single neuron level.

## Introduction

Recording from single neurons and stimulation to same microelectrodes near simultaneously is highly desirable for both basic neuroscience research and neural engineering applications. In electrophysiological studies, same electrode recording and stimulation would enable stimulus-response experiments at single neuron or small neuronal population level (Shepherd et al., 2001; Houweling and Brecht, 2008; Krause et al., 2019). In deep brain stimulation (DBS), which provides therapy to various neurological diseases such as movement disorder (Ackermans et al., 2006; Voges et al., 2007), depression (Schlaepfer et al., 2014), and epilepsy (Halpern et al., 2008), such technique would allow delicate micro-manipulation of complex neural circuits and monitoring feedback neural signals with high spatial resolution (Vesper et al., 2002; Little et al., 2013; Priori et al., 2013; Salam et al., 2016; Swan et al., 2018). In cortical prostheses such as the hippocampal memory prosthesis, which aims to restore cognitive functions by replacing damaged brain regions (Song et al., 2007, 2009; Berger et al., 2011; Hampson et al., 2018), stimulating and recording from the same single neurons becomes vital for successful implementation of the single neuron-level, multi-input, and multi-output model-based microstimulation (Deadwyler et al., 2018; Song et al., 2018).

All of these require high spatial resolution, high signal-to-noise ratio, feedback signals recorded from the stimulated tissue, power efficiency, and electrode stability. For recording, high spatial resolution and signal-to-noise ratio are necessary for differentiating single neuron activities from background noise. For stimulation, high spatial resolution is essential for focal delivery of electrical charge to the target neural tissue. Feedback control based on recording from the stimulated tissue enables proper adjustment of stimulation parameters over time. This is especially crucial in chronic implants where glial cell encapsulation can weaken the electrode-tissue interaction and cause reduction in the stimulation effect over time (Polikov et al., 2005). In addition, neural plasticity may alter response to stimulation, and make it necessary to use recorded feedback signals to optimize stimulation parameters (Kerr et al., 2011; Månsson et al., 2016). Lastly, free-roaming animal experiments and implantable neuromodulation devices both require low energy consumption and electrode stability for long-term use of the device.

These needs may be addressed with low-impedance microelectrodes that allow both stimulation and recording. The main challenge of such electrodes is that the geometric area (not accounting for surface roughness) of a recording electrode should be comparable to the size of a single neuron to record unitary activities, but at the same time, stimulation electrodes require relatively large surface area to obtain low electrochemical impedance that allows safe charge injection to evoke desired neural responses. In other words, reducing electrode size for high spatial resolution stimulation and recording generally results in an increase of the electrochemical impedance of the electrode-tissue interface (Cogan, 2008) and higher thermal noise (Suner et al., 2005). For stimulation electrodes, where the same amount of charge must be delivered across a smaller interface, the increased impedance results in increased electrode polarization, increased energy consumption, and limits maximum electrochemically reversible stimulation pulses (Merrill et al., 2005). It is therefore highly desirable to minimize electrochemical impedance of the electrode while keeping the electrode area small enough for single neuron recording.

The impedance of electrode/electrolyte interfaces is generally modeled as a combination of resistors and capacitance in parallel (Merrill et al., 2005). The simplest being the simplified Randles model, which consists of a resistor (representing the solution resistance) in series with a resistor (representing the charge transfer resistance) and a capacitor (representing the double layer capacitance) in parallel. Because impedance is proportional to resistance and inversely proportional to capacitance, efforts to decrease interface impedance are based on decreasing resistance and increasing capacitance.

Surface roughening is the most common method to increase the capacitance. By roughening the surface, electrochemical surface area is increased while the geometric (or macroscopic) surface area remains the same (Cogan, 2008). Platinum-black, a very friable coating, was originally used for surface roughening, which provoked severe foreign body response displacing neurons and disabling recording single neuron activities (Loeb et al., 1977). Other coatings that increase real surface area include Titanium Nitride (TiN) (Weiland et al., 2002), graphene oxide (Apollo et al., 2015), PEDOT (Boehler et al., 2019), and Carbon Nanotubes (CNT) (Wang et al., 2006; Baranauskas et al., 2011; Kozai et al., 2016).

To reduce resistance, either the electrolyte must be more conductive, or the charge transfer resistance must decrease. *In vivo*, the electrolyte is the tissue resistance. Strategies to decrease tissue resistance generally focus on reducing immune responses that can lead to fibrous encapsulation of the electrode (Polikov et al., 2005). These include reducing the size of the electrodes (Kozai et al., 2012), decreasing the mechanical mismatch between the electrode and the tissue (Kim et al., 2013; Luan et al., 2017; Xu et al., 2018; Wang et al., 2020), and incorporating bioactive molecules onto the surface of the electrode to attenuate the immune response (Zhong and Bellamkonda, 2007).

To reduce the charge transfer resistance, a valence-shifting layer that can absorb and desorb electrons and ions in a reversable manner can be incorporated into the electrode. Iridium oxide (IrOx), due to its multiple oxide states (Robblee et al., 1983), is the most common material with this property used in neural electrodes. Currently there are three approaches used to make IrOx coated microelectrodes: activated iridium oxide film where a bulk iridium electrode is oxidized by cycling it through positive and negative voltages in an aqueous solution (Beebe and Rose, 1988), sputtered iridium oxide films, where an iridium target is used in the presence of oxygen (Cogan et al., 2004b, 2009), and electrodeposited iridium oxide (Lu et al., 2009). These techniques also roughen the surface, thus increasing the capacitance as well. Among those approaches, electrodeposition has the unique advantage of being cost efficient as it does not require a cleanroom and can be selectively applied to any subset of biomedical electrodes within an array made from almost any electrically conductive material. Perhaps the biggest drawback to activated IrOx, however, is that it is a brittle material, which can cause it to fail when stimulating with high charge densities (Cogan et al., 2004a).

Electrodeposited Pt-Ir Coating (EPIC) is an electrodeposition process in which Pt and Ir are co-deposited onto a conductive surface. EPIC maintains the advantages of electrodeposited iridium oxide (increases surface area and lower charge transfer resistance), with the added benefit of containing Pt, a less brittle metal than Ir, which likely contributes to a more robust and less prone to delaminate coating compared to IrOx or PEDOT (Petrossians et al., 2011; Dalrymple et al., 2019; Welle, 2020). Pt-Ir has the added advantage of having been used (in one form or another) in FDA approved neuroelectronic devices for decades (Cogan, 2008), unlike TiN and PEDOT, which have only been used for pacemaker and cardiac mapping applications, respectively (Schaldach et al., 1989; Boehler et al., 2019), and graphene oxide and CNTs, which have not been used in FDA approved devices.

Electrodeposited platinum-iridium coating has demonstrated its ability to record single units through microelectrodes (Cassar et al., 2019), as well as deliver charge through relatively large cochlear electrodes both *in vitro* (Lee et al., 2018; Dalrymple et al., 2019) and *in vivo* (Dalrymple et al., 2020). Darymple et al. (Dalrymple et al., 2019) compared EPIC electrode to PEDOT and Graphene Oxide in an accelerated aging electrochemistry experiment in saline. The study concluded that PEDOT and Graphene Oxide coated electrodes exhibited an increase in impedance and reduction in charge storage capacity compared to EPIC coating after aging. In this study, EPIC was evaluated for its ability to enable stimulation through electrodes small enough to record single units. EPIC evaluation involved *in vitro* and acute *in vivo* electrochemical characterization including electrochemical impedance spectroscopy, cyclic voltammetry, and polarization waveform analysis.

In addition to *in vivo* electrochemical characterization, we acutely evaluated and compared the ability of EPIC coated and uncoated microelectrodes to bidirectionally stimulate to and record single units through the same microelectrode using an 8-channel microelectrode array implanted in the hippocampus with approximately every other electrode coated and the other half uncoated. Performance of coated electrodes was quantitatively compared with uncoated electrodes within the same device and across different animals. The electrodes were tested for signal-to-noise ratio, electrode polarization, energy efficiency, charge storage capacity, and capability to stimulate and record short latency and prolonged neural responses to various electrochemically reversable stimulation parameters.

Our results demonstrated that EPIC coating enabled electrochemically safe stimulation above 5 μA (>0.38 mC/cm^2^), which enabled recordings of spontaneous spikes, field excitatory post synaptic potentials (fEPSPs), and population spikes (PSs) from both the stimulation electrode and neighboring recording electrodes. Results further showed that, compared to uncoated electrodes, EPIC electrodes recorded neural signals with higher signal-to-noise ratios, and generated lower voltages for given stimuli. Thus, EPIC provides a powerful tool for monitoring and manipulating neural circuits at the single neuron level.

## Materials and Methods

### Electrode Arrays

**FIGURE 1:**
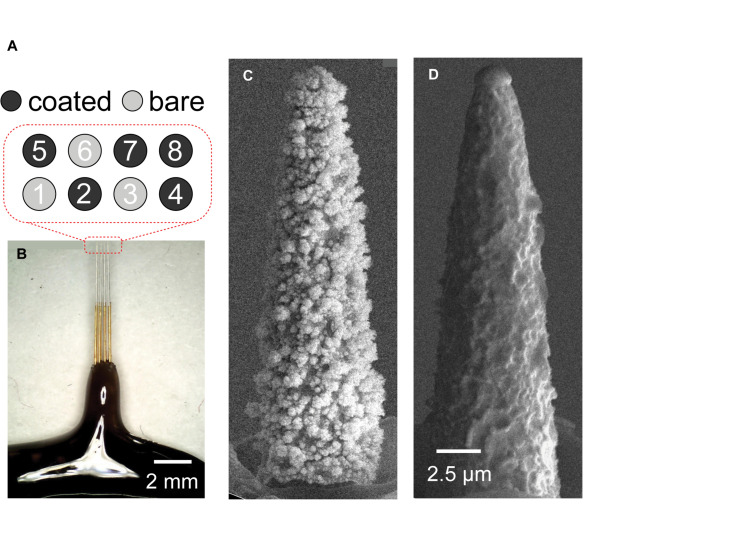
**(A)** Schematic showing relative locations of coated and uncoated contacts. This arrangement allowed a side-by-side comparison of contacts with and without Pt-Ir coating. **(B)** Optical micrograph of Microprobes microelectrode array. SEM micrograph of **(C)** Pt-Ir coated and, **(D)** uncoated microelectrode tips. **(C)** The highly porous nodular structure on the coated electrode dramatically increase the electrochemical surface area while the geometric surface area remains similar to **(D)** the uncoated electrode.

A commercially available, hand-made 8-channel microelectrode array (MEA) (Microprobes for Life Science, Gaitherbsurg MD; platinum-iridium, 6mm length, 75 μm diameter, 150 μm interelectrode spacing, ∼500 kΩ impedance) was used for this study. The device contained a 2 × 4 arrangement of Pt-Ir microelectrodes, expanding into an area that covers 300 μm × 750 μm. The entire length of each electrode was insulated with a layer of chemical vapor deposition of Parylene-C followed by another layer of polyimide tubing around the base of the electrodes for additional stiffness. The tip of each microelectrode was exposed by electropolishing Parylene-C to of approximately 10 μm in length for this study (Figure 1B). The final step of the electropolishing process is performed by passing current through each electrode to remove additional insulation until a specific impedance is met. Impedance measurements are noisy in microelectrodes, as is the case here. Therefore, this step introduces the main source of inter-electrode variability. By using the same device across animals, we have removed this major source of variability for better comparison between pre-implantation, *in vivo*, and post-implantation conditions.

The geometric surface area of the electrodes was approximated by the SEM image in Figure 1 using the formula for the surface area of a cone (height ∼ 20 μm; radius ∼ 3.5 μm) to be 2.6e-6 cm^2^. At the base, the microelectrodes were mated to a 10 channel Omnetics connector. The leads from the Omnetics connector were soldered onto a printed circuit board with a surface mounted header which split the leads out for easy connection using hook wires. The array was then electrochemically deposited with Pt-Ir (Epic Medical, Inc., Pasadena, CA) using a process described previously (Petrossians et al., 2011) resulting in 5 coated and 3 uncoated microelectrodes (Figure 1A).

Next the device was imaged with a scanning electron microscope (SEM). Imaging was performed using a field emission SEM (Joel JSM-7001) at 15 kV. SEM images of a coated and an uncoated electrode’s surface morphologies shown in Figures 1C,D, respectively, provided visual confirmation that the coating increased the effective area while maintaining the geometric area.

Prior and post every implantation, each microelectrode underwent electrochemical characterization in Phosphate Buffer Solution (PBS) including electrochemical impedance spectroscopy (EIS) (±10 mV vs. Ag| AgCl, 100 kHz – 0.5 Hz) and cyclic voltammetry (CV) (0.8 V to −0.6 V vs Ag| AgCl). It is standard practice to deoxygenate the solution for evaluating the electrochemical properties of materials. However, the focus of this particular study was to characterize the potential performance of the material for *in vivo* studies. Since *in vivo* conditions contain dissolved oxygen, running electrochemical tests in saline that has not been deoxygenated more closely matches the *in vivo* conditions. In addition, the electrochemical performance of iridium (in the Pt-Ir) rely on its reduction and oxidation and the presence of the O_2_ in the solution are part of those reactions.

Furthermore, voltage transient in response to single biphasic cathodic first current pulses were recorded across each microelectrode in 1/6 diluted PBS mimicking impedance of brain tissue of approximately 0.25 S/m (Kandadai et al., 2012) to determine the maximum electrochemically safe stimulation amplitudes given a fixed pulse duration of 200 μs. A polarization voltage of >−700 mV was considered safe for the cathodic phase and within water window (Cogan, 2008).

### Surgical Procedure

Electrochemistry and electrophysiology experiments were conducted in dorsal hippocampi of male Sprague-Dawley rats (*n* = 3, 350–450 g, 3–4 months). All procedures were performed in accordance with protocols approved by the Institutional Animal Care and use Committee of the University of Southern California. The rats were pre-anesthetized by an intraperitoneal injection of Ketamine and Xylazine cocktail. During the surgery, anesthesia was maintained with an inhalation of isoflurane (1∼2% in pure oxygen) administered through a nose cone from isoflurane machine. The status of anesthesia was checked frequently by pinching the toe or footpad, and a heating pad was used to maintain and monitor the animal temperature.

The animals were mounted onto the stereotaxic frame through ear bars. Craniotomy of 2 mm × 4 mm was performed over the right dorsal hippocampus. Dura and pia were removed before the implantation. The electrodes were inserted at ∼2.60 mm posterior to the bregma and ∼2.45 mm lateral to the midline, and it was angled ∼30 degrees from the midline to match the septal-temporal axis of the hippocampus. A micro-manipulator was employed to support and advance the electrode 2.5–3.8 mm from the surface of the cortex. A reference electrode was inserted far away from the electrode array in the hindbrain in each experiment.

Neural signals were monitored as the electrodes were advanced into the brain for the presence of complex spikes (a burst of 2–6 single spikes of decreasing amplitudes with < 5 ms interspike intervals (Ranck, 1973). Complex spikes serve as an electronic signature for pyramidal neurons of the hippocampus, which help confirm placement of the electrodes in the CA1 region of hippocampus. After the microelectrodes had reached the target location, data acquisition began.

Five sets of experiments were performed *in vivo*: (1) spontaneous activity recording, (2) *in vivo* EIS measurement, (3) recording of voltage transient response to stimulation, (4) stimulation and recording from the same and neighboring channels, (5) recordings from euthanized rat to separate neural responses from artifact and noise. Following each implantation, the electrodes were explanted and cleaned using cyclic voltammetry. Also, EIS measurements were taken to ensure that the impedance was not altered.

### Data Acquisition

All neural activities were digitized and recorded by a recording system (Digidata 1322A, Molecular Devices) and saved by pClamp9 (Molecular devices) software using 100 kHz sampling frequency. The recording amplifier was first set to a gain of 80 dB and a filter of 300 Hz–10 kHz to capture single unit activities. The output of the recording system was connected to a speaker to allow for auditory discrimination between single and complex spikes activity. The recording amplifier filter was then changed to a wideband filter of 1 Hz–10 kHz to capture single-unit as well as multi-unit activities.

### Spontaneous Activity Recording

Activities from two microelectrodes (one coated and one uncoated electrode) were simultaneously saved in one-minute long recordings for signal to noise ratio (SNR) analysis. The SNR was defined as the power spectral density (PSD) (averaged from 1 Hz to 5 kHz to include frequencies with associated power from multi and single units (Harrison, 2007) of the signal recorded from anesthetized rat (PSD_anesthetized_) divided by the PSD of recordings made from the same region after the rat was euthanized (PSD_euthanized_) (Suarez-Perez et al., 2018). Using PSD to calculate SNR eliminated the need for any assumptions about the amplitude of action potentials, as is necessary when SNR is calculated using a chosen threshold to separate noise from neural signal. PSD also allowed for a comparison of signal power from alive versus euthanized rats. The signal recorded from the euthanized rat is purely noise from the electrode and the recording system.

A mixed linear model was used to determine the statistical significance of coating on SNR. Independent *t*-test was applied to find out whether there is a significant difference between uncoated and coated microelectrodes within and across animals. All results are presented as mean ± standard error (SE).

### Electrochemical Characterization

Once spontaneous activity was recorded from all channels, the recording amplifiers were disconnected from the microelectrodes. Each contact was then connected one at a time to Gamry Reference 600 potentiostat (Gamry instruments, Warminster, PA, United States) to measure EIS. The faraday cage surrounding the surgery table was used as ground, and the Pt-Ir wire implanted in the hind brain as return electrode. The impedances as a function of frequency were plotted and compared between coated and uncoated electrodes.

### Stimulation Parameters

Next, the electrodes were connected to a custom-built stimulator, design of which was described elsewhere (Elyahoodayan et al., 2019). Charge-balanced, cathodic first, biphasic single pulses were delivered to the electrodes with each subsequent pulse having a larger total charge. A fixed pulse duration of 200 μs with no interface interval was used. Table 1 organizes the pulse parameters by duration, amplitude and total charge for coated and uncoated electrodes.

**TABLE 1:** List of stimulation test pulse parameters (pulse amplitude and total charge delivered).

Test #	Current (μA)	Charge (nC)	Charge density (mC/cm^2^)
1	1	0.2	0.077
2	2	0.4	0.153
3	3	0.6	0.230
4	4	0.8	0.308
5	5	1	0.385
6	10	2	0.770
7	20	4	1.54
8	30	6	2.31
9	40	8	3.08
10	50	10	3.85

*The same pulse duration (200 μs per phase) was used for all tests. Test numbers 1–5 were applied to both electrode types (coated and uncoated). Test numbers 6–10 (in gray) were only tested on the coated electrodes because the parameters exceeded safety limits when used on the uncoated electrodes. The uncoated electrode reached safety limits at 5 μA (CIC = 0.38 mC/cm^2^), whereas the coated electrode allowed a stimulation current of 50 μA (CIC = 3.85 mC/cm^2^) before reaching this limit.*

### Voltage Transient Response

The output of a previously designed stimulator PCB (Elyahoodayan et al., 2019) was connected to each electrode one at a time using a coaxial cable ending with hook cables. The voltage across the electrode in response to each stimulation pulse was digitized at 1 MHz sampling frequency and recorded. A duration of ∼1-s ground phase was used between each pulse to allow for complete discharge of the electrode before pulsing it with higher amplitude.

The maximum polarization in the cathodic phase across the electrode-tissue interface was calculated. There are two factors in the transient voltage response: the ohmic voltage drop (V_a_) arising from the ionic conductivity of the tissue (R_s_) and the polarization across the electrode-electrolyte interface (ΔE_p_). V_a_ and ΔE_p_ have some overlap due to small double layer capacitance of the uncoated microelectrodes, which introduces uncertainty into ΔE_p_ calculation. Another factor that contributes to this uncertainty arises from limitation of current sources when loaded with high impedance such is the case with microelectrodes. This limitation arises from an increased time constant at the output of the constant current stimulator. The resultant voltage response to the applied squared current pulses is a biphasic pulse with round corners, which makes clear ΔE_p_ measurements with microelectrodes more difficult.

**FIGURE 2:**
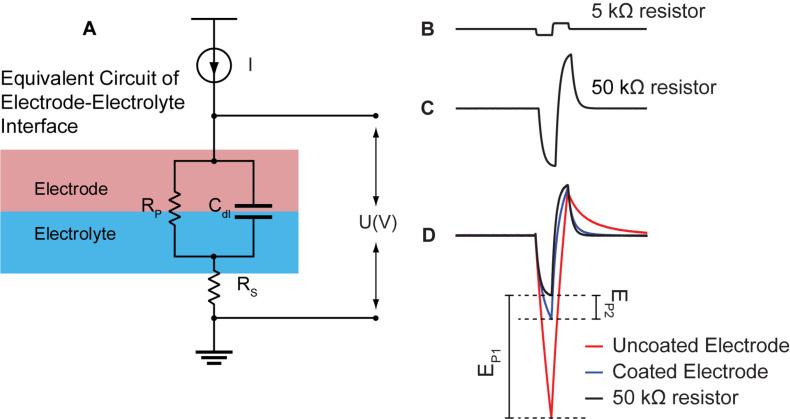
Quantification of electrode change in polarization from current pulsing. **(A)** Simplified electrical equivalent circuit model of the electrode-electrolyte interface. Electrode/electrolyte interface is modeled by a capacitor (C_dl_) and a resistor in parallel (R_p_). The resistance of the electrolyte is modeled using a simple resistor (R_s_). **(B)** Voltage response of constant biphasic pulse (with current I) sent through a single small (5 kΩ) resistor. There is a linear response that maintains the square wave of the biphasic pulse. **(C)** Voltage response to constant biphasic pulse sent through a single 50 kΩ resistor meant to approximate the R_s_ of tissue. Because of the size of the resistor, the voltage response is no longer linear likely due to the large time constant at the output of the stimulator. **(D)** The voltage response to the 50 kΩ resistor superimposed onto the voltage response of the coated (blue) and uncoated (red) electrodes. The polarization voltage for the uncoated electrode (E_p1_) and coated electrode (E_p2_) is the subtraction between V_a_ estimated in **(C)** and the voltage responses of the uncoated (red) and coated electrode (blue), respectively.

To mitigate these challenges, we calculated ΔE_p_ by (1) estimating R_s_ from EIS at >50 kHz, (2) recording voltage transient across an R_s_ equivalent, (3) subtracting the waveform obtained from an R_s_ equivalent from the electrode transient voltage waveform (Figure 2). All data are reported for the cathodic phase of the pulse as stimulation pulses are cathodic first and negative ΔE_p_ would be larger than positive ΔE_p_.

Next, energy consumption associated with driving current pulses through stimulation electrodes was computed using the equation below:

$$\mathrm{Power}=\int_{0}^{T}\mathrm{IV}\left(t\right)\mathrm{dt}$$

(Fink and Beaty, 1978), where V(t) is the transient voltage across the electrode; dt is the step size in time; T is the pulse duration; and I is the applied current to the electrode tissue interface. Energy consumption associated with driving the coated electrodes was compared to energy consumption used by uncoated electrodes to determine if any significant savings were gained by application of the coating.

An independent *t*-test was used to determine statistical significance of electrode polarization and energy consumption between coated and uncoated microelectrodes. All results presented as mean ± standard error (SE).

### Neural Response to Stimulation

Recording when used in conjunction with stimulation may cause prolong saturation of the recording amplifier, which would mask short latency neural response. Previously, we reported a stimulus artifact suppression technique that reduced the artifact down to ∼2 ms after the termination of the stimulation pulse from the stimulated electrode. The designed stimulus artifact suppression technique (Elyahoodayan et al., 2019) was used here to record short latency neural response to stimulation. In short, the design uses a set of CMOS switches to disconnect the electrodes from the recording amplifiers during stimulation. The ∼2 ms lag in recording after stimulation is because the artifact suppression technique is merely a suppression technique and does not completely remove the artifact to allow simultaneous recording and stimulation. It does, however, reduce the duration of the artifact.

The two-channel recording system was connected to the stimulating electrode and a neighboring electrode in the electrode array. With this arrangement, we could monitor the effect of stimulation on the targeted tissue and neighboring channels. Stimulation and recording were conducted twice using stimulation parameters summarized in Table 1 before switching the second channel of the recording system to another neighboring electrode. Thus, the stimulation electrode was pulsed 14 times using the same stimulation parameters. This experiment was repeated using an uncoated electrode as the stimulation electrode. A duration of ∼5-s recovery period was used to allow the tissue to return to base line before pulsing it with the next amplitude.

Directly evoked action potentials were recorded, and corresponding changes were observed in the multi-unit and single unit band, including an increase in magnitude of short latency evoked response and changes in spike rate associated with increasing stimulus amplitudes. Responses were recovered within 2.5 ms from the stimulating and neighboring electrode after the initiation of the stimulation pulses.

### Spike Sorting Analysis

Data from microelectrode recordings from the stimulation electrode before and after each stimulus were analyzed and activity of different neurons per microelectrode were identified by using Plexon off-line sorter. Since neuronal firing rates show temporal variability following stimulation, the time course of action potentials was shown as peri-event raster and peri-event histogram for each stimulation. Peri-event raster and histograms were initially visually inspected to identify firing patterns associated with stimulation. Successive trials were synchronized with the stimulation artifact for fEPSP responses.

## Results

### Electrochemical Measurements

**FIGURE 3:**
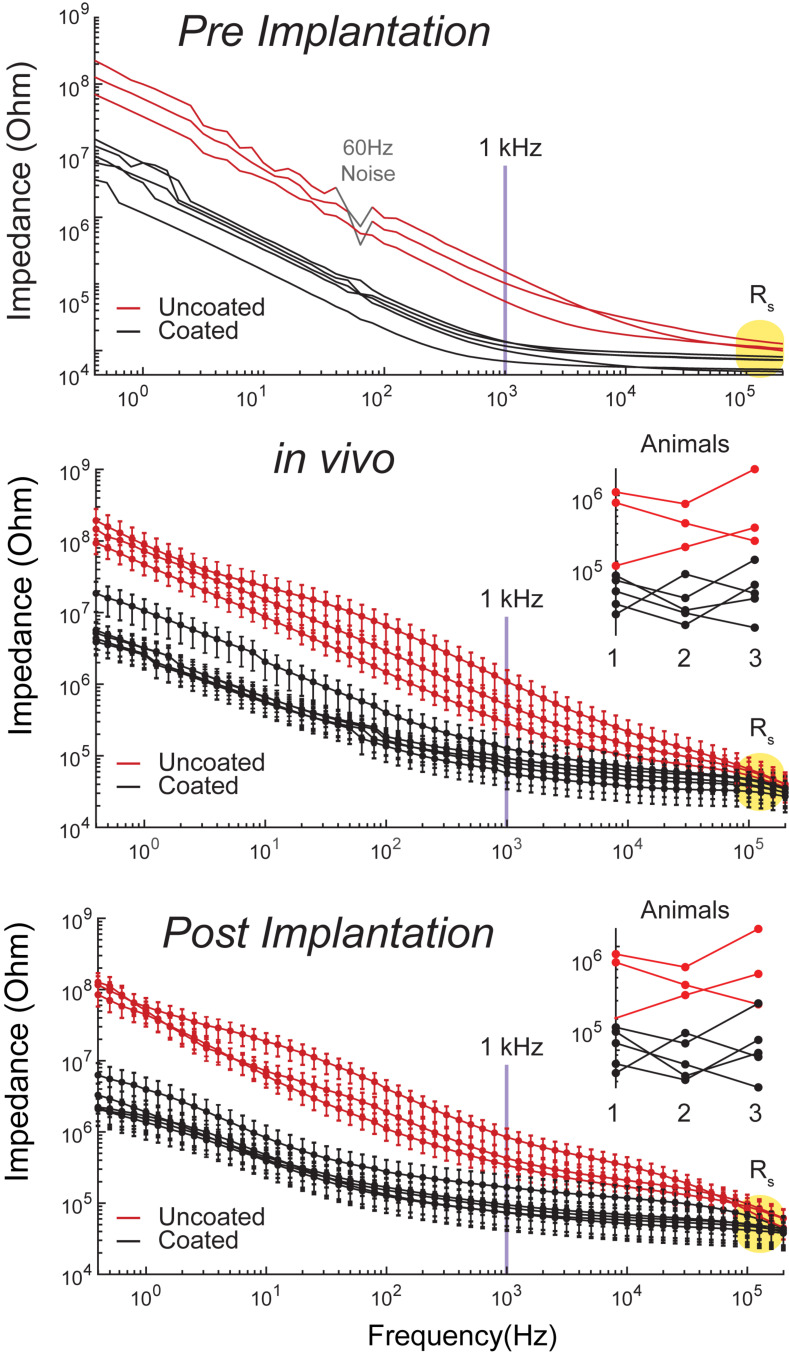
Bode plots of impedance magnitude | Z| vs. frequency recorded for coated (5 black traces) and uncoated (3 red traces) pre-implantation in room temperature PBS (top plot), in rat CA1 region of hippocampus (middle plot, *n* = 3 rats), and post-implantation in room temperature PBS (bottom plot, *n* = 3 rats). Impedance magnitude at 1 kHz demonstrated an average 8.4x× pre-implantation, 7.4× *in vivo*, and 7.8× post-implantation reduction. R_s_ is highlighted in yellow in all plots. Middle and bottom plot insets demonstrate that there is no consistent change in impedance arising from repeated use.

Electrochemical impedance spectroscopy data for 3 uncoated and 5 coated microelectrodes in room temperature PBS one time pre-implantation to assess baseline performance, as well as, in the CA1 region of hippocampus (*n* = 3), and in room temperature PBS after each implantation (*n* = 3) are shown in Figure 3 for comparison. The data is demonstrated in bode-plot format (phase angle not shown) in which the logarithm of the impedance is plotted as a function of the logarithm of frequency. Impedance from uncoated electrodes are noisy due to their high impedance. Furthermore, impedance *in vivo* is noisier due to high impedance of tissues in addition to high impedance of electrodes. At 1 kHz (center frequency of spike activity), the impedance for the electroplated microelectrode was reduced by approximately 8.4 × pre-implantation, and 7.4 × *in vivo*, and 7.8x post-implantation compared to that of the uncoated electrodes. The variabilities observed from 3 trials across all frequencies is very similar to each other because the change of electrolyte impedance causes a constant shift upward or downward in the overall impedance of the electrode-electrolyte interface.

At high frequencies (greater than approximately 500 kHz), impedance magnitudes showed resistive behavior representing R_s_. R_s_ is approximately 50 ± 9 kΩ *in vivo*, which is the value used to estimate V_a_ This is on average a 10 × difference in R_s_, causing an upward shift of the traces by a decade in the *in vivo* plots compared to the *in vitro* plots. Furthermore, R_s_ is inversely proportional to the exposed surface area of the electrode and the solution conductivity constant (Newman, 1965). Thus, the variability observed between electrode impedances both *in vitro* and *in vivo* is due to the variability in the electropolishing process to expose the electrodes’ tips by the manufacturer. Another source of variability *in vivo* is inhomogeneity in tissue resistivity causing larger spread across traces in comparison to the *in vitro* traces. Our results demonstrate that there is small variability across the coated electrodes as seen in Figure 3, where the distribution of the coated electrodes is similar to the uncoated electrodes. This is consistent with previous report on the same coating technology (Cassar et al., 2019).

The EIS plots in Figure 3 do not show significant changes in impedance from pre and post implantation, which indicates insignificant changes in electrode morphology pre and post implantation. This is consistent with previous reports on the same coating technology, where the EPIC coated electrodes were analyzed by SEM before and after chronic implantation with chronic stimulation (Dalrymple et al., 2020). Furthermore, Figure 3 middle and bottom plot insets demonstrate that there is no consistent change in impedance, which means that the variations do not arise from changes in Pt-Ir coating from repeated use, but are rather associated with other variables such as tissue impedance.

**FIGURE 4:**
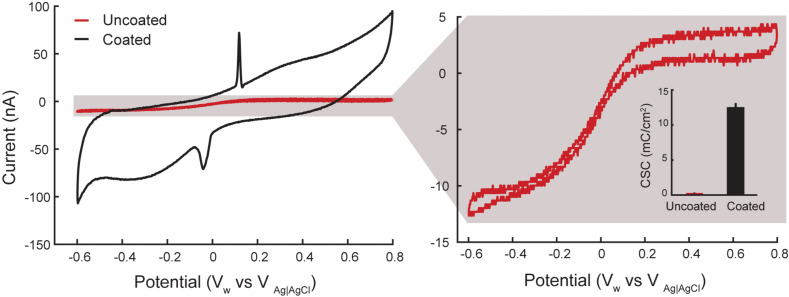
Representative cyclic voltammograms from rat 2 for coated (black) and uncoated (red) electrodes in room temperature PBS. Artifacts from silver leakage caused during the coating process are grayed out. The calculated CSC_C_ is an average of 0.3 ± 0.025 mC/cm^2^ for uncoated (*n* = 9 measurements from 3 electrodes in 3 rats) and 12.5+0.75 mC/cm^2^ for the coated (*n* = 15 measurements from 5 electrodes in 3 rats) electrodes.

Figure 4 displays a representative voltammogram of a coated and uncoated electrode in room temperature PBS. The zoomed inset signal in Figure 4 (right side) is noisy because of the high impedance of the electrodes and the small scale used compared to the uncoated electrodes. The cathodic capacity of the coated and uncoated electrodes was calculated from anodic to cathodic sweeps (100 mV/s) of the cyclic voltammetry (Merrill et al., 2005; Cogan, 2008). The coated electrodes drew 50 ± 3 nC (*n* = 10) and the uncoated electrodes drew 1.2 ± 0.1 nC (*n* = 6). Dividing the geometric surface area of the electrodes approximated from the SEM image by the measured capacity provides the cathodic charge storage capacitance (CSC_c_), which is defined as the total amount of charge available per geometric surface area for an electrode (Cogan, 2008). The calculated CSC_c_ was 12.5+0.75 mC/cm^2^ and 0.3 ± 0.025 mC/cm^2^ for the coated and uncoated electrodes, respectively. Thus, the coated electrodes generated a significantly higher current than uncoated electrodes (two-sample *t*-test *p* < 0.001), likely due to lower impedance of the coated electrodes.

The artifact corresponding to peaks on the CV plot of the coated electrode in Figure 4 at 0.175 V (during anodic sweep) and -0.05 V (during cathodic sweep) is likely from silver nanoparticle contamination during the coating process. This contamination may occur when some silver nanoparticles from the reference electrode leaks into the plating solution and bond to the electrodes surface. A detailed discussion regarding silver peaks in CV plots is discussed in (Van der Horst et al., 2015).

### Electrode Response to Stimulation Pulses

**FIGURE 5:**
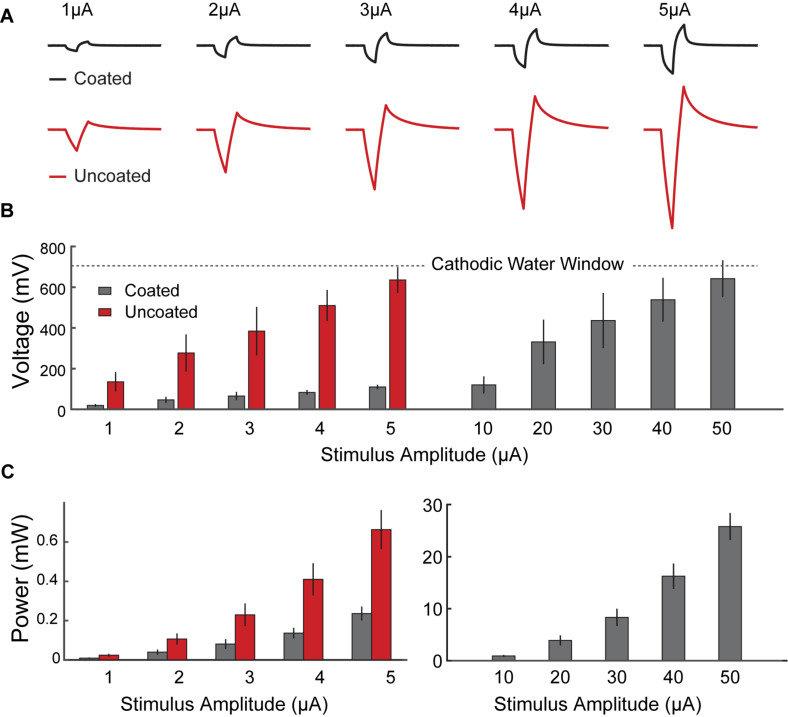
*In vivo* voltage transient of coated vs. uncoated electrodes, in response to biphasic current pulsing in rat hippocampus with respect to a PtIr reference electrode. **(A)** Representative voltage response traces from rat 2 recorded from coated (black) and uncoated (red) electrodes, in response to biphasic current pulses (pulse duration of 200 μs, amplitudes of 1 μA to 5 μA). **(B)** Average electrode’s change in polarization as a function of stimulus current pulses across the coated (black) and uncoated (red) electrodes. The uncoated electrode surface potential crosses the cathodic potential safety limit (*U* = –700 mV) at ∼5 μA, whereas the coated electrodes reached the same polarization in response to a 10 × larger current pulse (*I* = ∼50 μA). On average there is an 83% reduction in electrode polarization for all stimuli. **(C)** Calculated average energy consumption associated with driving the electrodes with biphasic stimulation pulses, plotted as a function of stimulus current magnitudes. Data comparing energy consumption in coated (gray) and uncoated (red) electrodes are shown on the left. Higher stimulation pulses applied to the coated electrodes are shown on the right. Error bars indicate standard error calculated for coated (*n* = 15 measurements from 3 electrodes in 3 rats) and uncoated (*n* = 9 measurements from 3 electrodes in 3 rats) electrodes. There is a statistically significant improvement in energy consumption using the coated electrodes (*p* < 0.001). On average there is 64% reduction in energy consumption across all stimuli.

Figure 5A shows representative voltage transient response curves at the stimulation electrode surface in response to cathodic first current pulses of 1 μA to 5 μA. After 200 μs the applied current was reversed by an equal but opposite anodic pulse, resulting in voltage transient in the positive direction. The voltage transient across the uncoated electrode resulted in polarization curves with masked V_a_ (R_s_ and E_p_ segments were merged). Generally, the voltage transient of the coated electrode showed smaller increase in voltage over the pulse interval, and the shape of E_p_ was more linear and less parabolic.

Figures 5B,C show the calculated E_p_ and energy consumption, respectively, plotted as a function of pulse current amplitude. Data from 1 μA to 5 μA are shown as comparison between the coated and uncoated electrodes. Data from 10 μA to 50 μA are shown for the coated electrodes only.

For E_p_, a conservative water window of −700 mV was chosen to avoid electrode potential exertion which is reached at the charge injection capacity (CIC) of the electrode (Cogan, 2008). The uncoated electrode reached this window at 5 μA (CIC = 0.38 mC/cm^2^), whereas the coated electrode allowed a stimulation current of 50 μA (CIC = 3.85 mC/cm^2^) before reaching this limit.

Consistently, for all five test pulses used, the coated electrodes showed a significantly lower E_p_, and energy consumption, as compared to the uncoated electrodes (*p* < 0.001 for all test cases). In all scenarios tested, the coated electrodes resulted an average 83% improvement in E_p_ and 64% improvement in energy consumption versus the uncoated electrodes. In chronic stimulation applications, this could lead to having stable electrodes and significant energy consumption savings.

### Recording of Spontaneous Neural Activity

**FIGURE 6:**
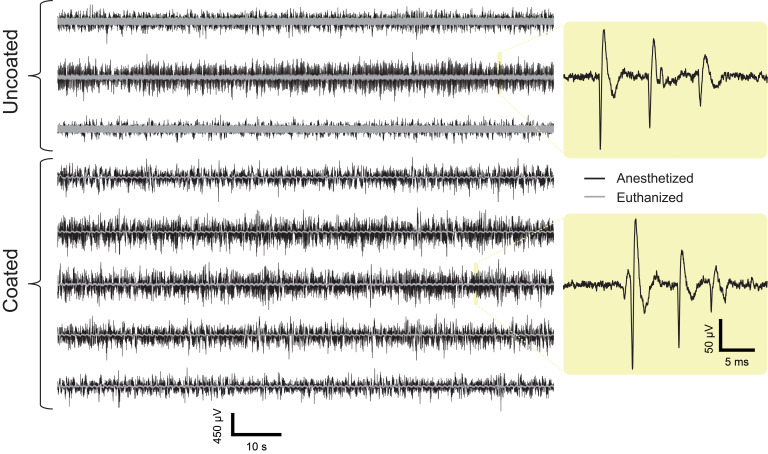
Comparison of spontaneous recordings made from uncoated (top 3) and coated (bottom 5) electrodes in the CA1 region of the hippocampus (all recordings are plotted on the same scale). Superimposed gray traces are recordings made from the same electrodes after the animal has been confirmed euthanized. Zoomed insets (right) are representative examples of complex spikes indicating proper placement of electrodes in the rat 3 CA1 region of hippocampus (euthanized recordings are omitted). The scale bar is for both coated and uncoated electrodes.

A total of 1-min representative sample plots of the signal recorded from an anesthetized rat overlaid with the signal recorded from a euthanized rat (considered to be the baseline noise of the system) are shown in Figure 6. The plot shows visual comparison of the signal and noise level of the coated and uncoated electrodes. It is apparent that the uncoated electrodes manifested higher noise level than the coated electrodes. A sample of complex spikes recorded from a coated and an uncoated microelectrode is shown in Figure 6 to demonstrate proper placement of the electrode array in the CA1 region of hippocampus.

**FIGURE 7:**
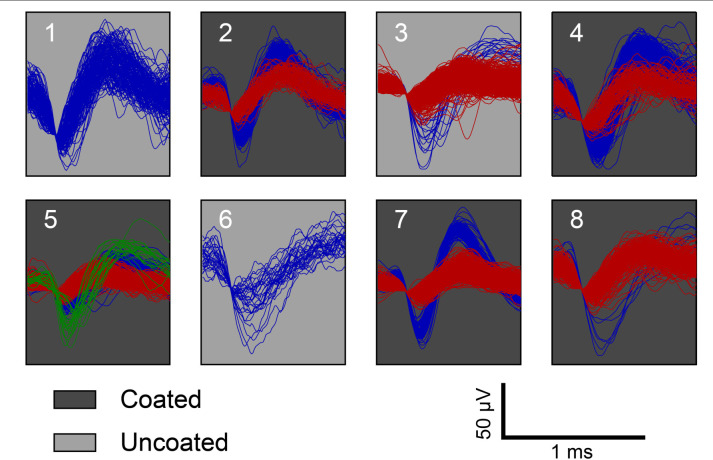
Representative example of acute single unit recordings in rat 3 from each electrode. Red, blue, and green traces represent different single units. White and gray background indicate coated and uncoated electrodes, respectively.

From the recording, single units were isolated at each time point and the number of discernable units per electrode was quantified for each electrode within the array (Figure 7). Figure 7 shows there is no significant amplitude difference between the coated and uncoated electrodes. Many factors contribute to the spike amplitude recorded from the electrode. Different neurons generate spikes with different amplitudes. Even for the same neuron, recording from soma and dendrites will produce large differences in spike shape and amplitude. In addition, the spike amplitude decays with distance. Electrodes with lower impedance will reduce thermal noise, but it is of marginal importance, since the 40-70 μV fluctuation caused by signaling units is several-fold higher for neurons close to the electrode (Boehler et al., 2020). Cassar et al. (2019) also reported amplitude differences between the coated and uncoated electrodes are likely unrelated to the impedance reduction from the coating. Hence in this study, spike amplitude is not used to compare the recording performance between the coated and uncoated electrodes.

**FIGURE 8:**
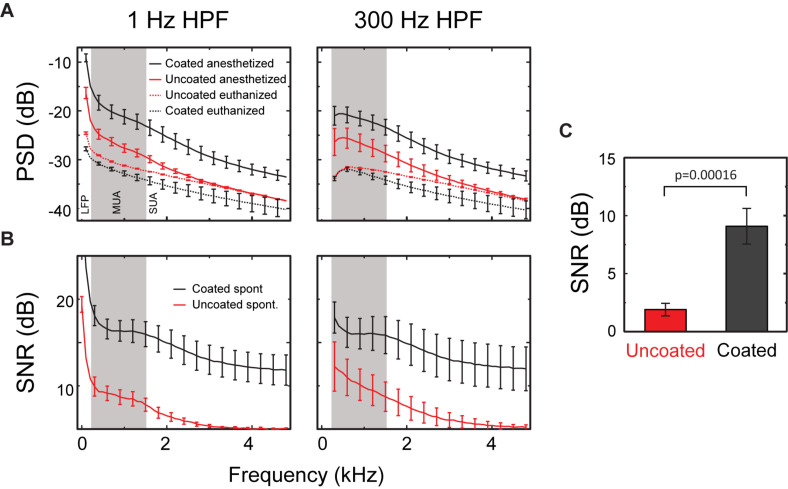
SNR of spontaneous activity. **(A)** PSD from 1 to 5 kHz of spontaneous neural recordings in the CA1 region of rat hippocampus (PSD_anesthetized_) and activity after the animal was euthanized (PSD_euthanized_) using either a 1 Hz HPF (left) or 300 Hz HPF (right). **(B)** The SNR from 1 to 5 kHz for uncoated (red) and coated (black) electrodes for recordings made with a 1 Hz HPF (left) or a 300 Hz HPF (right). SNR approaches 1 dB for frequencies above ∼3 kHz for uncoated electrodes when using a 1 Hz HPF and above 4 kHz when using a 300 Hz HPF. All results presented as mean ± SE for uncoated (*n* = 6 measurements from 3 electrodes in rats 2 and 3) and coated (*n* = 10 measurements from 5 electrodes in rats 2 and 3) electrodes. **(C)** Mean SNR calculated from neural recording data (300 Hz–5 kHz filtered) for uncoated (*n* = 12) and coated (*n* = 20) electrodes. The effect of the coating was statistically significant in the mixed linear model (*p* < 0.0002), but the effect of the filter was not (*p* = 0.81).

The difference in noise is illustrated when comparing the traces from the euthanized animal (Figure 6, gray traces). The uncoated electrodes illustrate a nosier signal level in the absent of any neural activity, so this noise is presumably there even when it is measuring from living tissue. A linear mixed model was used to determine the statistical significance of the coating and the filter on PSD’s calculated from PSD_anesthetized_ and, PSD_euthanized_. Because the same electrodes were used with different rats, the animal was included as a random effect. The linear model had 3 significant effects (Figure 8A). (1) As expected, PSD_euthanized_ was significantly lower than PSD_anesthetized_ [χ^2^(1) = 67.95, *p* = 2.2e-16], which validated our decision to use these recordings as our baseline noise for SNR calculations. (2) For all frequencies, PSD_anesthetized_ was significantly higher in the coated electrodes compared to uncoated electrodes (χ^2^(1) = 3.87, *p* = 0.049). (3) PSD_euthanized_ was significantly lower in the uncoated electrodes compared to coated electrodes [χ^2^(1) = 14.35, *p* = 0.00015] (in contrast to the opposite relationship for spontaneous activity described in effect number 2). This can be seen in Figure 8A in the PSD values especially near -40 dB for PSD_euthanized_ made from coated electrodes. When comparing the PSD_euthanized_ versus the PSD_anesthetized_ traces for coated electrodes, there is a difference in magnitude greater than the standard error across all frequencies (Figure 8A). In contrast, the difference between PSD_euthanized_ and PSD_anesthetized_ for the uncoated electrodes is smaller and approaches zero with increasing frequency.

It is worth noting that the uncoated electrodes exhibit an increase in their baseline noise compared to the coated electrodes which is visually observed in the gray traces of Figure 6. However, the increase in the amplitude of neural activity in the coated electrodes compared to the uncoated electrodes is less obvious in Figure 6. This is because the recorded neural signal amplitude is dependent on which neuron the electrode is recording from, as well as, the distance of the electrode to the firing neurons. However, the linear model found a significant difference between the coated and uncoated population of PSDs.

Signal to noise ratio was defined as PSD_anesthetized_ – PSD_euthanized_. A linear model with the coating and filter as fixed variables and animal as a random variable was fit to the SNR data (Figure 8B). The results of the linear model showed a significant effect of the coating [χ^2^(1) = 14.2, *p* < 0.00016], with coated microelectrodes having higher SNR (coated = 9.09 ± 1.53 dB, uncoated = 1.90 ± 0.50 dB, Figure 8C). The filter used (1 Hz or 300 Hz) did not have a significant effect on PSD [χ^2^(1) = 1.57, *p* = 0.21]. Thus, Figure 8 shows that the PSD for the coated electrodes is higher than the uncoated electrodes at all frequencies, and the difference between the PSD_anesthetized_ and PSD_euthanized_ is larger at higher frequencies for the coated arrays.

### Short Latency Neural Response to Stimulation

**FIGURE 9:**
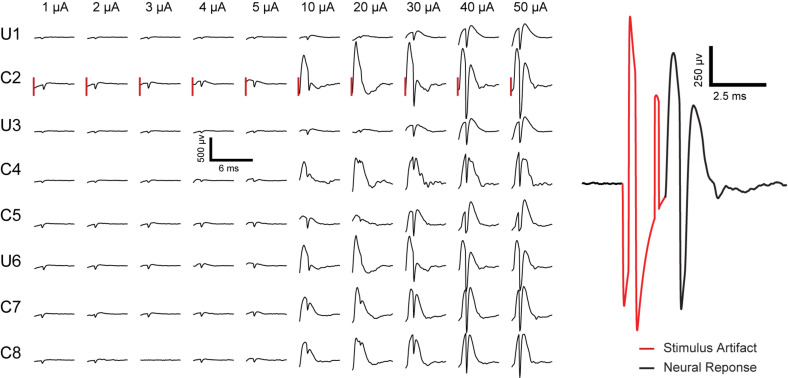
Representative electrically evoked fEPSP and PS in rat 3 recorded from all eight electrodes inresponse to biphasic current pulsing through electrode # 2 in anesthetized rat CA1 region of hippocampus. “U” and “C” written on the left side of the plot represent the uncoated and coated electrodes, respectively, followed by the electrode number (electrode configuration as sketched in Figure 1A). The vertical lines to the left of each response for C2 represent a time 2.5 ms after the initiation of the stimulation pulse (pulse amplitude labeled above each line). Stimulus pulse amplitudes of 1 μA to 5 μA induced potentiation of PS without fEPSP. Pulse amplitudes of 10 μA to 50 μA cause potentiation of PS accompanied with fEPSP. An example of stimulus artifact (red) followed by neural response (EPSP and PS) is presented on the right.

Short latency extracellular evoked responses were obtained from the CA1 cell body layers following stimulation. Here, stimulation is through one electrode and recording is from all electrodes (stimulating electrode plus the other electrodes). A total of 80 response curves were generated to monitor changes 2.5 ms following the initiation of 10 separate stimulation pulses across a coated stimulation electrode and recordings across all 8 electrodes. The results in Figure 9 can be separated into two categories depending on whether only PSs were potentiated or PSs plus fEPSP were potentiated. At low amplitudes (1–5 μA), PSs are potentiated in the absence of fEPSP. At amplitudes above 10 μA, potentiation of PSs was accompanied by potentiation of fEPSP.

**FIGURE 10:**
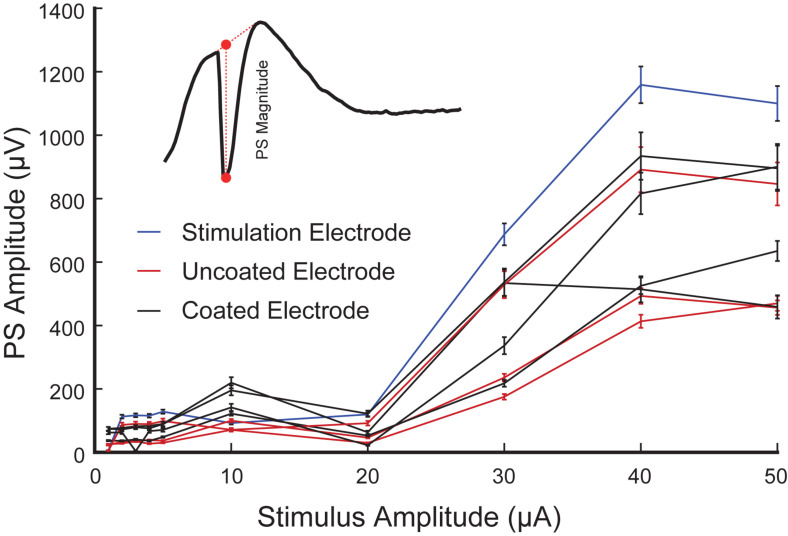
Representative input-output curves of PS amplitude in the CA1 region of rat hippocampus (4 trials from rat 3 are included in the analysis). PS amplitude corresponding to the stimulation electrode (blue), and the neighboring coated (black) and uncoated (red) electrodes are shown. Inset is a schematic diagram of a PS (negative trough) accompanied by an fEPSP (positive hump). The PS amplitude is computed as the difference between the nadir of the PS and the interpolated value of the fEPSP at that same time (as shown in red dash line).

The amplitude of PS was measured as the difference in voltage between the nadir of the PS trough and the mean in voltage between the fEPSP peaks on either side of the negative deflection (Figure 10 inset) (Gholmieh et al., 2004). The input-output response curves were generated using 1–50 μA stimulation amplitudes. Statistics were performed on raw values of PS amplitude determined from average waveforms (4 trials from the same animal are included in the analysis). When only PS was potentiated there was almost no change in the recorded amplitude with increasing stimulation amplitude. In contrast, when fEPSP plus PS were potentiated the calculated amplitude increased with the stimulus amplitude and saturated at 40 μA (Figure 10).

It is important to note that neural response to stimulation is dependent on factors such as distance from the stimulating electrode, tissue anatomy, and distance of firing neuron to recording electrode. As the focus of this paper is *in vivo* evaluation of electrodeposited microelectrodes, further neuroscientific analysis of the neural response will be discussed in future studies.

### Prolong Effect of Stimulation

**FIGURE 11:**
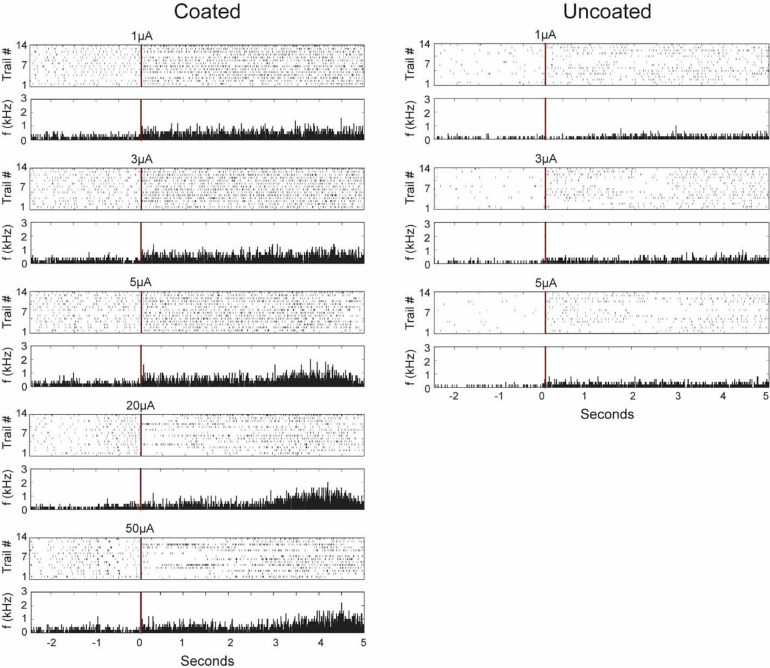
Representative neuronal firing patterns before and after stimulation from the coated (left) and uncoated (right) electrode recorded from the stimulation electrode in the CA1 region of rat 3 hippocampus neuron. The red vertical lines represent the time of stimulation with its corresponding magnitude written above it. The plots consist of peri-event raster and its corresponding peri-event histogram below it. Each dot in the raster plot represents the occurrence of a single action potential in the recorded neuron for 14 trials in the same animal. The peri-histogram represents spike counts accumulated per 5 ms bins.

Figure 11 shows characteristics of neurons recorded from the stimulation electrode (coated and uncoated) presenting each firing pattern 14 times before and after selected stimulation pulses as a peri-event raster. From the raster plot corresponding to the coated electrode, we found two type of responses to stimulation: excitation only and inhibition-excitation. Excitation only activity demonstrates an increase in firing rate proceeding stimulation at amplitudes below 5 μA. Inhibition-excitation happens at and above 10 μA, which triggers activation of interneurons followed by excitation. A surprising finding is the long wave of inhibition in some trials (not all) of up to 1.5 s, followed by excitation, which may have clinical and pathophysiological implications not yet understood. From the uncoated electrode, excitation-only activity was observed as the electrode was limited to low amplitude stimulation pulses for safety. In both cases, there are some similarities and variabilities across different trials apparent within a stimulus in the raster plot which may be resulting from stimulating a dynamic neurophysiological mechanism.

The neuronal responses to electrical stimulation were also classified based on peri-stimulus time histogram (PSTH). The response patterns were clustered using 50 ms bin intervals and averaged across 14 trials. Initial inhibition observed in some trials in the raster plot is masked in the PSTH as there seems to be an increase in spike rate in other trials. However, it is clear from the PSTH that there is an increase in spike rate at around 3 s post stimulation observed from the coated electrode.

Regarding electrode impedance and spikes rate, Figure 7 shows that there is no significant difference in unit yield between coated and uncoated electrodes. Electrodes with lower impedance have a better recording performance due to reduced thermal noise. As long as neurons are clearly distinguishable above the noise floor, electrodes measured the same overall number of units for coated and uncoated electrodes (Boehler et al., 2020). Cassar et al. (2019) also reported the unit yield of the coated and uncoated electrodes began at approximately the same level (Cassar et al., 2019), which is consistent with the acute recording presented in this manuscript. Different level of spike firing rate is an intrinsic property of neurons and is independent of electrode impedance. Also, there is a high degree of variability in neuron firing rate and firing rate changes between coated and uncoated electrodes cannot be concluded. Hence in this study, unit yield and spike firing rate are not used to compare the recording performance between the coated and uncoated electrodes.

This study demonstrated that EPIC coating enabled observation of short latency (∼2.5 ms) and long latency neural response to electrochemically safe stimulation pulses of above 3.85 mC/cm^2^. Figure 11 demonstrates varying neural responses were generated by increasing the stimulation current. Conventional microelectrodes cannot produce such results because of limitations in charge injection capacitance and strong stimulation artifact masking short latency neural response to stimulation.

## Discussion

In neural modulation/prosthetic systems, it is essential to understand the effect of stimulation and electrophysiological responses involved in the brain. There are two types of responses. (1) Immediate response shown in Figure 9 which demonstrates different evoke response may be elicited in response to stimuli with varying amplitude. (2) Delayed modulatory effect shown in Figure 11, which demonstrates modulation of firing pattern and rate in individual neurons. These results are critical for understanding the effect of electrical stimulation at different intensities and further enables researchers to optimize stimulation parameters.

Closed-loop DBS has been performed and exhibited promise in many previous studies, where stimulation parameters are adjusted based on feedback from neural activities (Lutz et al., 2013; Priori et al., 2013; Little et al., 2016; Salam et al., 2016). However, in these studies the recording electrodes were separate from stimulation electrodes. In a study by Tabot et al. (2013) stimulation and recording from the same electrode were performed to restore sense of touch using IrOx coated microelectrodes. However, single unit recording is not reported, and stimulation and recording is done serially as the recording equipment is first hooked up to run an experiment followed by disconnecting the recording set-up and hooking up the stimulation equipment. Zhou et al. (2019) demonstrated near simultaneous stimulation and recording through microelectrodes; however, LFPs and no single units are reported. It is also not clear whether the stimulation and recording is occurring on the same contact or through two nearby contacts. To the best of our knowledge, no previous study has demonstrated stimulation in parallel with recording of single unit activities as well as evoked responses in response to stimulation from the same microelectrode.

In this study, we quantified the performance of microelectrodes in an array electroplated with Pt-Ir when used for stimulation and recording on the same electrode and further compared them with uncoated microelectrodes on the same array. Results showed that coated electrodes exhibited superior performance compared with uncoated electrodes in terms of SNR, energy consumption, electrode polarization, and charge storage capacitance. Quantitative analysis indicated substantial improvements of coated electrodes over uncoated electrodes due to reduction of impedance.

Lower electrochemical impedance magnitude of a microelectrode improves recording performance by reducing thermal noise and thereby increasing SNR. Here, we evaluated SNR by recording from each electrode before and after the animal was euthanized. The signals recorded after euthanasia were considered noise arising from the electrode-tissue interface and the recording system. Since the same recording system was used to record from each electrode, the only variable contributing to noise was the electrode. We then quantified SNR using power spectral density analysis, which demonstrated statistically significant improvement. Results showed that lower impedance of coated electrodes extended to above 1 kHz, which is the frequency range of single-unit and multi-unit activities and LFP’s that are biomarkers in closed-loop neuromodulation.

Minimizing electrode impedance is highly desirable in chronic neuro-stimulation applications as our results suggested that coated electrodes exhibited higher energy efficiency and lower electrode polarization. Improved energy efficiency and polarization voltage are due to the fact that energy and voltage are directly proportional to electrode impedance. Improved energy efficiency is essential in free-roaming battery-powered animal experiments and battery-operated implantable neural modulation/prosthetic systems (Berger et al., 2005, 2012; Song and Berger, 2013; Miranda et al., 2015; Lo et al., 2017; Zhou et al., 2019). Improved polarization voltage results in long-term stability of the electrode because continuous high polarization at the electrode/electrolyte interface can lead to dissolution (McHardy et al., 1980), corrosion (Schuettler and Stieglitz, 2002), and/or deformation of the electrode (Ordonez et al., 2015). Furthermore, our results show that the coated microelectrodes have on average 41 × of the viable charge for the same geometric surface area.

However, since CSC_c_ is obtained under low sweep rates and low current densities, it is not an accurate measure of safe reversable charge injection capacities of the electrode during stimulation with constant current biphasic pulses. To assess this parameter, we applied biphasic pulses with a constant duration and increased the stimulation amplitude until the electrode polarization crossed a predefined water window of −700 mV. What we observed with the uncoated electrodes was that the voltage transient response exhibited a masked R_s_ response with visible asymmetry of the biphasic pulses. R_s_ response is masked because of limitations of the current source being loaded with high impedance, which increases the rise time at the output of the current source and causes round edges in response to a square pulse. Furthermore, asymmetry is a result of a small double layer capacitor in the electrical equivalent circuit of the electrode-electrolyte interface, which dominated the transient response over the response due to R_s_. On the other hand, the coated microelectrodes exhibited a typical transient response recorded from macroelectrodes such as the ones used in DBS. Overall, the transient voltage from coated microelectrodes were lower with faster discharge period due to symmetry in biphasic pulses.

To obtain a more accurate estimate of the polarization voltage, we applied the same biphasic pulses across a resistor that mimicked R_s_ and subtracted the waveform from the transient voltage response across electrodes. What we found was that the coated electrodes allowed 10× of the stimulation amplitude compared to the uncoated electrodes. Subsequently, more charge per phase of stimulation pulse may be applied to the microelectrode without causing irreversible reactions. This corresponded to a charge injection capacitance of 3.8 mC/cm^2^ for the coated electrodes. Widening the range of stimulation parameters is especially valuable in chronic applications where adjustment, typically an increase, of stimulation parameters is needed over time due to changes in neural circuitry as well as the electrode-tissue interface.

It is important to note that in general the electrochemical impedance magnitude of the coated microelectrodes is reduced as result of increasing the effective area of the electrode and not the geometric surface area. Therefore, the coated microelectrodes could inject a larger range of reversable stimulation pulses to the tissue while maintaining the ability to record single unit activity as shown in Figure 7.

In conclusion, EPIC coating allowed us to use microelectrodes designed for single unit recording as stimulation electrodes. We demonstrated this capability in immediate and prolonged neural responses to stimulations by recording fEPSPs, PSs, and spontaneous spikes from the same and neighboring microelectrodes in response to varying stimulation parameters. Thus, EPIC coated microelectrodes offer the capability of closed-loop neural stimulation to and recording from the same microelectrodes and provides a powerful tool for monitoring and manipulating neural circuits at the single neuron level.

## Data Availability Statement

The original contributions presented in the study are included in the article/supplementary material, further inquiries can be directed to the corresponding author/s.

## Ethics Statement

The animal study was reviewed and approved by Institutional Animal Care and use Committee of the University of Southern California.

## Author Contributions

SE, JW, CL, AP, and DS contributed in electrode design and design of animal studies. JW, GW, and AP contributed in Pt-It coating of electrodes. SE, JW, CL, and AP contributed in bench-top electrode characterization. WJ contributed in surgical procedures. WJ, SE, and AP contributed in *in vivo* electrochemistry and electrophysiology experimentation. CL, AP, WJ, AS, SE, and DS contributed in data processing and analysis. All authors contributed to manuscript revision, read and approved the submitted version.

## Conflict of Interest

CL, GW, and AP were employed by the company Epic Medical, Inc. (now EPIC Medical). AP and JW are founders of the company Epic Medical, Inc. The remaining authors declare that the research was conducted in the absence of any commercial or financial relationships that could be construed as a potential conflict of interest. The authors declare that this study received funding from Epic Medical, Inc. The funder had the following involvement with the study: purchased the electrode for the study (∼$500 per array), provided coatings for the study free of charge, and partially funded the research efforts of author Sahar Elyahoodayan in the form of payments to USC ($8100). As stated previously, multiple authors are associated with Epic Medical, Inc., and as such contributed to the study design, data analysis, decision to publish, and preparation of the manuscript.
